# Cellular Tolerance Induced by Chronic Opioids in the Central Nervous System

**DOI:** 10.3389/fnsys.2022.937126

**Published:** 2022-06-28

**Authors:** Sweta Adhikary, John T. Williams

**Affiliations:** Vollum Institute, Oregon Health & Science University, Portland, OR, United States

**Keywords:** opioid, tolerance, electrophysiology, kinases, withdrawal

## Abstract

Opioids are powerful analgesics that elicit acute antinociceptive effects through their action the mu opioid receptor (MOR). However opioids are ineffective for chronic pain management, in part because continuous activation of MORs induces adaptive changes at the receptor level and downstream signaling molecules. These adaptations include a decrease in receptor-effector coupling and changes to second messenger systems that can counteract the persistent activation of MORs by opioid agonists. Homeostatic regulation of MORs and downstream signaling cascades are viewed as precursors to developing tolerance. However, despite numerous studies identifying crucial mechanisms that contribute to opioid tolerance, no single regulatory mechanism that governs tolerance in at the cellular and systems level has been identified. Opioid tolerance is a multifaceted process that involves both individual neurons that contain MORs and neuronal circuits that undergo adaptations following continuous MOR activation. The most proximal event is the agonist/receptor interaction leading to acute cellular actions. This review discusses our understanding of mechanisms that mediate cellular tolerance after chronic opioid treatment that, in part, is mediated by agonist/receptor interaction acutely.

## Introduction

Mu opioid receptor (MOR) ligands are the first choice for the treatment of acute, post-surgical or trauma. There are however side effects that limit their utility including respiratory depression, constipation sedation, dizziness, and nausea; chronic: abuse potential, dependence (Paul et al., 2021). Treatment with opioids have limited value for long-term treatment of most chronic pain. The development of analgesic tolerance is one component limiting the value of chronic treatment with opioids. A second component results from the development of opioid use disorder. Given the widespread distribution of MORs in the central nervous system (CNS), it is not surprising that multiple systems level actions are associated with both the acute and chronic actions of opioids. MOR expressing areas directly involved in the pain pathway include primary afferent, dorsal horn, and thalamic neurons (reviewed, Corder et al., 2018). There are also multiple pain associated regions that express MORs, such as the parabrachial area, periaqueductal gray, and rostral ventromedial medulla (reviewed, Corder et al., 2018). Additionally, actions of MORs in limbic areas such as the ventral tegmental area, nucleus accumbens, medial striatum, and rostromedial tegmental nucleus underlie the initial processes in the development of tolerance and consequently opioid abuse disorder (reviewed, Williams et al., 2013). The effects of opioids therefore result from actions in multiple areas at both the pre- and postsynaptic levels. Further complicating this, the downstream receptor-dependent signaling cascades vary across brain regions. Receptor actions are defined in part by expression levels, the complement of downstream effectors and the efficiency of receptor/effector coupling. Postsynaptic actions include inhibition mediated by an increase in potassium conductance and an inhibition of voltage dependent calcium channels and adenylyl cyclase (reviewed, Williams et al., 2001). In addition, receptors located on presynaptic terminals act to inhibit transmitter release. Opioid receptor dependent inhibition of GABA and glutamate results in the modulation of postsynaptic neurons through disinhibition and inhibition, respectively. Recent interest in agonist bias of G protein verses arrestin activation across different neurons has added another important layer in the understanding of the downstream actions of opioids (Gillis et al., 2020; Stahl and Bohn, 2021). A substantial component of opioid tolerance results from the downstream adaptations that result from continued MOR signaling during chronic treatment. These processes counteract continued MOR signaling also underlie the withdrawal that results following termination of opioid treatment. This review will discuss two levels of tolerance, namely receptor dependent and systems dependent tolerance.

Although the acute actions of opioids are established in multiple CNS areas, there are few areas where the mechanisms that underlie tolerance and the adaptive mechanisms that result from chronic treatment have been examined. It is also important to distinguish acute desensitization from long-term tolerance. Opioid signaling is disrupted in both processes, but there are distinct differences. Acute desensitization is most often induced with high concentrations of agonist that results in a reduction of signaling. Acute desensitization develops in minutes and recovers in 10’s of minutes upon agonist removal (reviewed, Williams et al., 2013; Birdsong and Williams, 2020). It is established that phosphorylation of the C-terminus of MOR is a necessary step in the induction of acute desensitization. Recent work indicates that acute desensitization is largely blocked by inhibition of GRK2/3 adding to work indicating a key role for protein kinase C (PKC) (Bailey et al., 2006) and c-Jun N-terminal Kinase (JNK) (Melief et al., 2010).

Tolerance to opioids, on the other hand, requires treatment with agonist for hours or days and is not associated with measurable change in MOR mRNA or protein expression (Ammon-Treiber and Hollt, 2005; Dang and Christie, 2012). The recovery from tolerance is very slow (days–months). Further the time course of this recovery is dependent on what measure is used to determine tolerance implying that tolerance is cell type and/or pathway selective (reviewed, Williams et al., 2013). Similar to acute desensitization, components of long-term tolerance are also dependent on phosphorylation of the C-terminus, in that cellular tolerance is blocked with the expression of receptors where phosphorylation sites in the C-terminus are mutated to alanine (Arttamangkul et al., 2018; Kliewer et al., 2019). There are however downstream adaptive mechanisms at the cellular level that result from the continued activation of receptors that persist in the absence of C-terminus phosphorylation (Kuhar et al., 2015; Leff et al., 2020; Adhikary et al., 2022a). The goal of this review is to summarize what is known about the development of tolerance in single neurons induced by chronic treatment with opioids. This cell-centric view of opioid actions is the basis of circuit and systems level outcomes following chronic opioid treatment and a full-appreciation of cellular changes across relevant brain regions will be critical in the search for ligands that can provide efficacious analgesia over extended periods without untoward actions on other circuits.

## Areas Where Neurons Have Been Examined Following Chronic Morphine Treatment

Morphine, a partial MOR agonist, remains a gold-standard for acute pain management despite a number of averse potential outcomes. Postsynaptic tolerance measured in brain slices from animals exposed to chronic morphine treatment has been examined using several protocols. The classical method to describe receptor tolerance requires concentration response curve in preparations from opioid naïve and treated animals. Early work in brain slices of rat locus coeruleus (LC) determined the concentration response to [D-Ala^2^, *N*-MePhe^4^, Gly-ol]-enkephalin (DAMGO) and normorphine by measuring the activation of potassium conductance (Christie et al., 1987). In slices from morphine treated animals there was a twofold rightward shift in the DAMGO, a full agonist, concentration response curve and the maximum current induced by normorphine, a partial agonist, was decreased to ∼50% of that measured in slices from untreated animals (Christie et al., 1987). Additionally, the decreases in response to both DAMGO and normorphine was long lasting (6 h). The interpretation of this result was that a reduction in receptor reserve caused the decrease in potassium conductance induced by a saturating concentration of ligand in preparations from morphine treated animals compared to naïve animals. Thus, measuring potassium conductance using partial agonists provide a sensitive assay to determine the decrease in receptor reserve. More recent results found two components to cellular tolerance induced by chronic morphine treatment. One transient form of cellular tolerance declined as the circulating concentration of morphine (1 μM) washed out of the brain slice over 60–90 min and was sensitive to inhibition of PKC (Bailey et al., 2009; Levitt and Williams, 2012). The transient decrease in signaling was considered to be a form of desensitization that recovered with the removal of morphine. Long-term tolerance as previously reported persisted for at least 6 h following preparation of the brain slice (Christie et al., 1987; Levitt and Williams, 2012). The results carried out in the LC from mice were similar but not identical in that the degree of tolerance induced by morphine in the mouse LC is qualitatively less than that measured in rat (Quillinan et al., 2011). There is also a difference in signs of acute withdrawal between mice and rat indicating a marked species difference in the adaptive processes following chronic treatment with opioids (Uddin et al., 2021).

Recent results carried out in rat brain slices in the Kölliker-Fuse (KF) – a region involved with respiratory control – indicate that the degree of tolerance induced after chronic (6–7 days, 80 mg/kg/day) morphine treatment was very small (Levitt and Williams, 2018). Conversely, in dissociated PAG and primary afferent neurons from mouse, the action of DAMGO to inhibit voltage dependent calcium current was reduced in preparations from morphine treated animals (Connor et al., 2015). This result differs from those obtained in brain slices of the LC where the maximum current induced by DAMGO, a potent and efficacious agonist, was the same in preparations from control and morphine treated animals (Christie et al., 1987; Connor et al., 2015). The difference between these results can be explained by the differences in receptor reserve (as defined by the number of receptors and/or the receptor/effector coupling efficiency) in the LC versus dissociated PAG neurons. The activation of potassium conductance in acutely isolated LC neurons, where the dendritic arbor was eliminated, was reduced relative to that in brain slice preparations (Ingram et al., 1997). Further, morphine was unable to activate the potassium current and blocked the current induced by [Met]5enkephalin (ME) or DAMGO. The interpretation was that morphine occupied MORs, but the receptor/effector coupling was reduced to the point that potassium channels were not activated. Distinct differences between cell types have also been characterized in experiments using AtT20 cells (Miess et al., 2018). With the combination of whole cell or perforated patch recordings to examine acute morphine dependent desensitization, there was no desensitization with whole cell recordings that was present with perforated patch recordings. The interpretation was that whole cell recording resulted in a washout of a soluble cellular component that was necessary for acute desensitization (Miess et al., 2018). Thus, the measurement of opioid induced tolerance is highly dependent on both the cell under study and the method used to obtain the results even when examining the same cell type. Despite these complexities, the data generated to date suggests that the degree of cellular tolerance measured using the activation of potassium conductance or the inhibition of voltage gated calcium channels in single neurons is species, cell type, and brain region dependent.

## Postsynaptic Adaptive Mechanisms

Beyond the decrease in downstream effector activation, chronic morphine treatment also affects MOR-regulating processes. For example, acute desensitization of the MOR is more pronounced after chronic morphine and methadone treatment (Dang and Williams, 2004, 2005; Quillinan et al., 2011; Arttamangkul et al., 2018). Additionally, the recovery from acute desensitization of MORs is prolonged following chronic treatment with morphine and the kinase regulation of G protein coupled receptors (GPCRs) is altered (Quillinan et al., 2011; Arttamangkul et al., 2018; Leff et al., 2020).

Opioid induced acute desensitization in the LC has been shown to be primarily homologous, in that desensitization of the MOR does not affect signaling of another GPCR on the same cell (Harris and Williams, 1991; Bailey et al., 2003, 2009; Dang et al., 2011). A decrease in sensitivity following chronic opioid treatment is also restricted to MORs and not to other GPCRs that couple to the same effectors (Christie et al., 1987; Connor et al., 1999; Bailey et al., 2009), suggesting specific actions on MOR and not on downstream effectors such as G-protein activated inwardly rectifying potassium channels that carry the described potassium conductance. However, multiple G_*i/o*_ coupled GPCR in the LC share signaling components, and there is evidence for heterologous desensitization to the α_2_ adrenergic receptor after MOR activation in mouse LC based on a more sensitive assay (Dang et al., 2012). In that study the current induced by a low concentration of noradrenaline was compared before and following acute desensitization induced by ME and a component of heterologous desensitization was detected. Furthermore, heterologous desensitization of the α_2_ adrenergic receptor was also shown in rats less than 20 days old in the LC (Llorente et al., 2012). Finally recent work found that chronic morphine treatment disrupted the ability of the GPCR kinase G protein coupled receptor kinase (GRK2/3) blocker, compound 101, to inhibit the recovery from MOR desensitization as well as the acute desensitization of the somatostatin receptor (Leff et al., 2020).

The mechanism underlying increased desensitization and slowed recovery from desensitization after chronic morphine treatment is phosphorylation dependent. In animals expressing total phosphorylation deficient (TPD) MORs, acute desensitization of MORs is blocked and the recovery from desensitization is faster compared to WT animals chronically treated with morphine (80 mg/kg/day, Arttamangkul et al., 2018). The kinase that is mainly responsible for acute desensitization of MOR is the GPCR kinase, GRK2/3, and blockade of GRK2/3 can nearly abolish desensitization (Doll et al., 2012; Lowe et al., 2015; Miess et al., 2018). However, inhibition of GRK2/3 after chronic morphine treatment was no longer sufficient to block desensitization or recovery from desensitization (Leff et al., 2020). Additionally, inhibitors of kinases including GRK2/3, PKC, and JNK were required to block desensitization, suggesting that chronic morphine treatment led to adaptations that induced functional adaptations of other kinases (Leff et al., 2020).

## Presynaptic Adaptive Mechanisms

Tolerance to opioids measured at the presynaptic level has been examined for decades beginning with early studies with the guinea pig ileum and mouse vas deferens. Following chronic morphine treatment there was a rightward shift in the concentration response curve that resulted from a reduction in MOR receptor reserve (Chavkin and Goldstein, 1984). This study was the prelude to others that indicated that a reduction in receptor reserve may be a common mechanism that underlies cellular tolerance. Although it is possible that there is a reduction in receptor number, a more likely explanation is that there is a decrease in the receptor/effector coupling.

Acute regulation of MORs upon agonist binding in the presynaptic terminal region of neurons is generally thought to utilize different mechanism than postsynaptic receptors. One key difference is that no acute desensitization was detected following application of a saturating concentration of agonist (Blanchet and Lüscher, 2002; Fyfe et al., 2010; Pennock et al., 2012; Jullié et al., 2020). The mechanisms underlying this lack of desensitization are not fully known, but recent work using single particle tracking has demonstrated that presynaptic MORs are phosphorylated and internalized, but are rapidly replaced at sites of transmitter release by lateral diffusion of extrasynaptic axonal receptors (Jullié et al., 2020). The extrasynaptic MORs were not subject to phosphorylation or internalization such that they are poised to replenish receptors at the sites of transmitter release.

One hallmark of downstream adaptive mechanism following long-term opioid exposure is the compensatory upregulation of adenylyl cyclase (Sharma et al., 1975; Terwilliger et al., 1991; Avidor-Reiss et al., 1995). The functional consequence of the increase in adenylyl cyclase activity is an over recovery of cAMP-dependent processes that remained in the continued presence of opioids (Sharma et al., 1975). Upon removal of morphine there was a marked overshoot in the production of cAMP and was implicated in a cellular form of acute opioid withdrawal. The role of adenylyl cyclase following chronic morphine treatment has been examined at multiple synapses (Bonci and Williams, 1996, 1997; Chieng and Williams, 1998; Ingram et al., 1997; Vaughan et al., 1997; Shoji et al., 1999). The increase in cAMP production has two downstream consequences. First is increased activation of PKA that augments transmitter release (Bonci and Williams, 1996, 1997; Chieng and Williams, 1998; Ingram et al., 1998) and through the addition of opioid sensitive adenylyl cyclase increased the inhibition mediated by opioids upon withdrawal (Ingram et al., 1998). Second, cAMP is metabolized in the extracellular space to adenosine (Brundege et al., 1997). The increase in extracellular adenosine then acts on adenosine A1 receptors to decrease transmitter release (Matsui et al., 2014). This modulation of adenosine by opioids is synapse specific (Brundege and Williams, 2002a) and could be dependent on the location of adenosine release in a given synapse (Adhikary et al., 2022b). Additionally, chronic morphine treatment can also increase the sensitivity of adenosine to A1 receptors (Brundege and Williams, 2002b). The increase in transmitter release in the continued presence of opioids is viewed as an adaptive mechanism that counters opioid induced inhibition of release and represents a form of cellular tolerance. Upon withdrawal of opioids the rise in extracellular adenosine to depress transmitter release is thought to represent a mechanism that reduces the signs of acute opioid withdrawal.

## Adaptive Mechanisms Following Chronic Treatment With Agonists of Varying Potency and Efficacy

It is established that different agonists induce distinct patterns of analgesic tolerance *in vivo*. By reducing the number of functional receptors with an irreversible antagonist of MORs (β-chlornaltrexamine, β-CNA), the analgesic efficacy in the whole animal was determined measuring the antinociceptive effect a number of opioids after partial irreversible antagonism. High-efficacy agonists require fewer receptors to produce antinociception and are therefore less affected by partial irreversible block with β-CNA, than the antinociceptive response for low-efficacy agonists (Kumar et al., 2008; Madia et al., 2009; Sirohi et al., 2009). These studies have found that fentanyl has the greatest relative efficacy, followed by etorphine, methadone and morphine, hydromorphone, oxycodone, and lastly hydrocodone. Relative efficacy also correlated with analgesic tolerance with low-efficacy agonists like morphine and oxycodone inducing greater tolerance more rapidly than high-efficacy agonists like etorphine and fentanyl (Walker and Young, 2001; Grecksch et al., 2006; Pawar et al., 2007; Kumar et al., 2008). Additionally, high dose etorphine, but not morphine or oxycodone, induced a substantial upregulation of dynamin-2, leading to downregulation of MORs (Pawar et al., 2007).

The mechanism underlying agonist specific *in vivo* tolerance is largely unknown, however, work in brain slice experiments from LC neurons have found that opioid agonists with different potencies and efficacies exert unique their effects on the MOR activation and regulation (Virk and Williams, 2008; Quillinan et al., 2011; Adhikary et al., 2022a). In rats treated with morphine, the acute decline of peak current by ME and morphine was facilitated and recovery from desensitization was reduced compared to untreated animals (Dang and Williams, 2004, 2005). The enhancement of desensitization suggests that after chronic treatment a subsequent desensitizing stimulus causes a greater uncoupling of MORs from its effectors compared to untreated animals. Rats chronically treated with methadone also had increased desensitization, and the concentration-response curve of ME was right-shifted twofold, but the recovery from desensitization was the same as in untreated animals (Quillinan et al., 2011). In experiments with rats chronically treated with oxycodone there was no rightward shift in the concentration-response curve to ME or oxycodone. There was also no change in the extent of desensitization or the rate of recovery from desensitization (Adhikary et al., 2022a). There was a rightward shift in the concentration response to ME in rats treated with fentanyl and in increase in the extent of desensitization (Adhikary et al., 2022a). These data support a critical role of agonist efficacy in mediating cellular tolerance after chronic treatment.

It is important to note that, the induction of tolerance to morphine on single cells in the LC required sustained treatment. Animals treated for 1 day with morphine did not exhibit any form or tolerance nor was the recovery from acute desensitization affected (Quillinan et al., 2011). In addition, the decrease in the rate and extent of recovery from acute desensitization in slices taken from morphine treated animals was not dependent on the dose of morphine applied using the osmotic mini pump (Quillinan et al., 2011). The conclusion is that continued signaling, even at a low level, was required to induce tolerance to morphine. Although the same result was not induced by chronic treatment with methadone, unlike treatment with morphine, it is possible that tolerance to methadone requires more than one week.

Cellular tolerance as measured by upregulation of second messengers also is agonist specific. Chronic morphine treatment resulted in a functional upregulation of PKC and JNK, resulting in these kinases contributing to desensitization of MOR and Somatostatin receptors (Leff et al., 2020). Curiously, even without inducing changes to MOR desensitization and tolerance, chronic oxycodone treatment (30 mg/kg/day) resulted in changes in the kinase dependence of somatostatin receptor desensitization (Adhikary et al., 2022a). Thus the continued signaling of MOR by oxycodone induced an adaptation downstream that altered the desensitization of somatostatin receptors. One possible explanation is that persistent MOR signaling with agonists that do induce desensitization or internalization had cellular effects unrelated to receptor dependent tolerance. This observation was based on experiments where animals were treated with fentanyl, a highly efficacious internalizing agonist. In those experiments, tolerance was measured by measuring a rightward shift in the concentration response curve to ME and demonstrated that chronic treatment with fentanyl (1.5 mg/kg/day) induced tolerance at the receptor level but did not cause an alteration in the kinase regulation of the somatostatin (SST) receptor (Adhikary et al., 2022a).

The role of phosphorylation of the C terminus induced by fentanyl after chronic treatment was examined with experiments using the expression of MORs where all phosphorylation sites on the C terminus were mutated to alanine (TPD-MORs). Treatment of animals with fentanyl expressing the TPD-MORs resulted in an altered kinase regulation of the somatostatin receptor, unlike experiments with wild-type MORs (Adhikary et al., 2022a). The experiment supported the role of continued signaling as a key mechanism that underlies the regulation of kinase dependent desensitization of GPCRs. Equally possible is that receptor dependent desensitization and internalization prevents the induction of altered kinase regulation of GPCRs by a downstream mechanism unrelated to acute signaling. The precise mechanisms that underly the induction of receptor dependent tolerance and adaptations that affect downstream processes at the cellular level are not known. It is however clear that the two processes are agonist dependent in the LC.

## Conclusion

Ultimately, how different agonists mediate regulation of MORs after chronic treatment, and therefore, the combination of receptor and cellular dependent tolerance are not fully understood. It is also not known if agonist efficacy or some other regulatory property of an agonist plays a role in mediating tolerance at the level of the receptor. For example, tolerance is induced by chronic treatment with morphine in spite of the fact that it is relatively inefficient at inducing desensitization and internalization. The idea that cellular tolerance is dependent on internalization was suggested by experiments where, morphine-bound MORs on the plasma membrane were phosphorylated and presumed desensitized (Zhang et al., 1998; Koch et al., 2001, 2005). Therefore, one theory of tolerance postulates that the lack of internalization, and consequently reduced recovery from desensitization, contributes to tolerance. A second theory states that the lack of internalization induced by morphine leads to continuous and persistent signaling, resulting in counter regulatory adaptations (Whistler and von Zastrow, 1998; Whistler et al., 1999). It is possible that both persistent signaling and decoupling of MORs from effectors contribute to cellular tolerance but it is clear that tolerance measured at the cellular level is only one component of the tolerance that is measured in living animals. The future understanding of tolerance will require work that connects cellular tolerance at the cellular and synaptic level in single neurons with whole animal work. What is known in the LC is a start but a complete understanding can only be accomplished through cellular and synaptic work in multiple areas of the CNS. Synapse specific effects of acute opioid actions are underway (Birdsong et al., 2019), but there is much to be done.

## Author Contributions

All authors listed have made a substantial, direct, and intellectual contribution to the work, and approved it for publication.

## Conflict of Interest

The authors declare that the research was conducted in the absence of any commercial or financial relationships that could be construed as a potential conflict of interest.

## Publisher’s Note

All claims expressed in this article are solely those of the authors and do not necessarily represent those of their affiliated organizations, or those of the publisher, the editors and the reviewers. Any product that may be evaluated in this article, or claim that may be made by its manufacturer, is not guaranteed or endorsed by the publisher.

## References

[B1] Adhikary S. Jaeckel E. R. Birdsong W. T. (2022a). Mu opioid receptors acutely regulate adenosine signaling in striatal glutamate afferents. *J. Neurosci.* 12 2404–2417. 10.1523/JNEUROSCI.1039-21.2022 35091505 PMC8944233

[B2] Adhikary S. Koita O. Lebowitz J. J. Birdsong W. T. Williams J. T. (2022b). Agonist specific regulation of G protein-coupled receptors after chronic opioid treatment. *Mol. Pharmacol.* 5 300–308. 10.1124/molpharm.121.000453 35193934 PMC9092468

[B3] Ammon-Treiber S. Hollt V. (2005). Morphine induced changes of gene expression in the brain. *Addict. Biol.* 10 81–89. 10.1080/13556210412331308994 15849022

[B4] Arttamangkul S. Heinz D. A. Bunzow J. R. Song X. Williams J. T. (2018). Cellular tolerance at the μ-opioid receptor is phosphorylation dependent. *Elife* 7:e343989. 10.7554/eLife.34989 29589831 PMC5873894

[B5] Avidor-Reiss T. Bayewitch M. Levy R. Matus-Leibovotch N. Nevo I. Vogel Z. (1995). Adenylyl cyclase supersensitization in mu-opioid receptor transfected Chinese hamster ovary cells following chronic opioid treatment. *J. Biol. Chem.* 50 29732–29738. 10.1074/jbc.270.50.29732 8530363

[B6] Bailey C. P. Kelly E. Henderson G. (2003). Protein kinase C activation enhances morphine-induced rapid desensitization of mu-opioid receptors in mature rat locus coeruleus neurons. *Mol. Pharmacol.* 66 1592–1598. 10.1124/mol.104.004747 15361548

[B7] Bailey C. P. Llorente J. Gabra B. H. Smith F. L. Dewey W. L. Kelly E. (2009). Role of protein kinase C and mu-opioid receptor (MOPr) desensitization in tolerance to morphine in rat locus coeruleus neurons. *Eur. J. Neurosci.* 29 307–318. 10.1111/j.1460-9568.2008.06573.x 19200236 PMC2695152

[B8] Bailey C. P. Smith F. L. Kelly E. Dewey W. L. Henderson G. (2006). How important is protein kinase C in mu-opioid receptor desensitization and morphine tolerance? *Trends Pharmacol. Sci.* 27 558–565. 10.1016/j.tips.2006.09.006 17000011

[B9] Birdsong W. T. Jongbloets B. C. Engeln K. A. Wang D. Scherrer G. Mao T. (2019). Synapse-specific opioid modulation of thalamocortico-striatal circuits. *Elife* 8:e45146. 10.7554/eLife.45146 31099753 PMC6541437

[B10] Birdsong W. T. Williams J. T. (2020). Recent progress in opioid research from an electrophysiological perspective. *Mol. Pharm* 98 401–409. 10.1124/mol.119.119040 32198208 PMC7562972

[B11] Blanchet C. Lüscher C. (2002). Desensitization of mu-opioid receptor-evoked potassium currents: initiation at the receptor, expression at the effector. *Proc. Natl. Acad. Sci. U.S.A* 99 4674–4679. 10.1073/pnas.072075399 11917119 PMC123706

[B12] Bonci A. Williams J. T. (1996). A common mechanism mediates long-term changes in synaptic transmission after chronic cocaine and morphine. *Neuron* 16 631–639. 10.1016/s0896-6273(00)80082-3 8785060

[B13] Bonci A. Williams J. T. (1997). Increased probability of GABA release during withdrawal from morphine. *J. Neurosci.* 2 796–803. 10.1523/JNEUROSCI.17-02-00796.1997 8987801 PMC6573250

[B14] Brundege J. M. Diao L. Proctor W. R. Dunwiddie T. V. (1997). The role of cyclic AMP as a precursor for extracellular adenosine in the rat hippocampus. *Neuropharmacology* 9 1201–1210. 10.1016/s0028-3908(97)00102-0 9364475

[B15] Brundege J. M. Williams J. T. (2002a). Differential modulation of nucleus accumbens synapses. *J. Neurophysiol.* 12 142–151. 10.1152/jn.00766.2001 12091540

[B16] Brundege J. M. Williams J. T. (2002b). Increase in adenosine sensitivity in the nucleus accumbens following chronic morphine treatment. *J. Neurophysiol.* 3 1369–1375. 10.1152/jn.00508.2001 11877511

[B17] Chavkin C. Goldstein A. (1984). Opioid receptor reserve in normal and morphine tolerant guinea pig ileum myenteric plexus. *Proc. Natl. Acad. Sci. U.S.A* 22 7253–7257. 10.1073/pnas.81.22.7253 6095280 PMC392117

[B18] Chieng B. Williams J. T. (1998). Increased opioid inhibition of GABA release in nucleus accumbens during morphine withdrawal. *J. Neurosci.* 17 7033–7039. 10.1523/JNEUROSCI.18-17-07033.1998 9712672 PMC6792981

[B19] Christie M. J. Williams J. T. North R. A. (1987). Cellular mechanisms of opioid tolerance: studies in single brain neurons. *Mol. Pharmacol.* 32 633–638. 2824980

[B20] Connor M. Bagley E. E. Chieng B. C. Christie M. J. (2015). β-Arrestin-2 knockout prevents development of cellular μ-opioid receptor tolerance but does not affect opioid-withdrawal-related adaptations in single PAG neurons. *Br. J. Pharmacol.* 172 492–500. 10.1111/bph.12673 24597632 PMC4292963

[B21] Connor M. Borgland S. L. Christie M. J. (1999). Continue morphine modulation of calcium channel currents in acutely isolated locus coeruleus neurons from morphine-dependent rats. *Br. J. Pharmacol.* 7 1561–1569. 10.1038/sj.bjp.0702922 10602337 PMC1571773

[B22] Corder G. Castro D. C. Bruchas M. R. Scherrer G. (2018). Endogenous and exogenous opioids in pain. *Ann. Rev. Neurosci.* 8 453–473. 10.1146/annurev-neuro-080317-061522 PMC6428583 29852083

[B23] Dang V. C. Chieng B. Azriel Y. Christie M. J. (2011). Cellular morphine tolerance produced by beta-arrestin-2-dependent impairment of mu opioid receptor resensitzation. *J. Neurosci.* 19 7122–7130. 10.1523/JNEUROSCI.5999-10.2011 21562274 PMC6703228

[B24] Dang V. C. Chieng B. Christie M. J. (2012). Prolonged stimulation of mu opioid receptors produces beta-arrestin-mediated heterologous desensitization of alpha2-adrenoceptor function in locus coeruleus neurons. *Mol. Pharmacol.* 3 473–480. 10.1124/mol.112.079350 22689562

[B25] Dang V. C. Christie M. J. (2012). Mechanisms of rapid opioid receptor desensitization, desensitization and tolerance in brain neurons. *Br. J. Pharmacol.* 165 1704–1716. 21564086 10.1111/j.1476-5381.2011.01482.x PMC3372824

[B26] Dang V. C. Williams J. T. (2004). Chronic morphine treatment reduces recovery from opioid desensitization. *J. Neurosci.* 24 7699–7706. 10.1523/JNEUROSCI.2499-04.2004 15342737 PMC3631536

[B27] Dang V. C. Williams J. T. (2005). Morphine-induced mu opioid receptor desensitization. *Mol. Pharmacol.* 4 1127–1132. 10.1124/mol.105.013185 16020743

[B28] Doll C. Pöll F. Peuker K. Loktev A. Glück L. Schulz S. (2012). Deciphering m-opioid receptor phosphorylation and dephosphorylation in HEK293 cells. *Br. J. Pharmacol.* 167 1259–1270. 10.1111/j.1476-5381.2012.02080.x 22725608 PMC3504992

[B29] Fyfe L. W. Cleary D. R. Macey T. A. Morgan M. M. Ingram S. L. (2010). Tolerance to the antinociceptive effect of morphine in the absence of short-term presynaptic desensitization in rat periaqueductal gray neurons. *J. Pharmacol. Exp. Ther.* 335 674–680. 10.1124/jpet.110.172643 20739455 PMC2993552

[B30] Gillis A. Gondin A. B. Kliewer A. Sanchez J. Lim H. D. Alamein C. (2020). Low intrinsic efficacy for G protein activation can explain the improved side effect profiles of new opioid agonists. *Sci. Signal.* 14:670. 10.1126/scisignal.aaz3140 32234959

[B31] Grecksch G. Bartzscj K. Widera A. Becker A. Hollt V. Koch T. (2006). Development of tolerance and sensitization to different opioid agonists in rats. *Psychopharmacology* 2 177–184. 10.1007/s00213-006-0365-8 16572262

[B32] Harris G. C. Williams J. T. (1991). Transient homologous mu-opioid receptor desensitization in rat locus cereus neurons. *J. Neurosci.* 8 2574–2581. 10.1523/JNEUROSCI.11-08-02574.1991 1651377 PMC6575524

[B33] Ingram S. Wilding T. J. McCleskey E. W. Williams J. T. (1997). Efficacy and kinetics of opioid action on acutely dissociated neurons. *Mol. Pharm.* 52 136–143. 10.1124/mol.52.1.136 9224823

[B34] Ingram S. L. Vaughan C. W. Bagley E. E. Connor M. Christie M. J. (1998). Enhanced opioid efficacy in opioid dependence is caused by an altered signal transduction pathway. *J. Neurosci.* 18 10269–10276. 10.1523/JNEUROSCI.18-24-10269.1998 9852564 PMC6793345

[B35] Jullié D. Stoeber M. Sibarita J. B. Zieger H. L. Bartol T. M. Arttamangkul S. (2020). A discrete presynaptic vesicle cycle for neuromodulator receptors. *Neuron* 105 663–677. 10.1016/j.neuron.2019.11.016 31837915 PMC7035187

[B36] Kliewer A. Schmiedel F. Sianati S. Bailey A. Bateman J. T. Levitt E. S. (2019). Phosphorylation-deficient G-protein-biased μ-opioid receptors improve analgesia and diminish tolerance but worsen opioid side effects. *Nat. Com.* 10:367. 10.1038/s41467-018-08162-1 30664663 PMC6341117

[B37] Koch T. Schulz S. Pfeiffer M. Klutzny M. Schroder H. Kahl E. (2001). C-terminal splice variants of the mouse mu-opioid receptor differ in morphine-induced internalization and receptor resensitization. *J. Biol. Chem.* 33 31408–31414. 10.1074/jbc.M100305200 11359768

[B38] Koch T. Widera A. Bartzsch K. Schulz S. Brandenburg L. O. Wundrack N. (2005). Receptor endocytosis counteracts the development of opioid tolerance. *Mol. Pharmacol.* 1 280–287. 10.1124/mol.104.004994 15475572

[B39] Kuhar J. R. Bedini A. Melief E. J. Chiu Y.-C. Striegel H. N. Chavkin C. (2015). Mu opioid receptor stimulation activates c-Jun N-terminal kinase distance arrestin-dependent and independent mechanisms. *Cell Signal.* 27 1799–1806. 10.1016/j.cellsig.2015.05.019 26056051 PMC4492890

[B40] Kumar P. Sunkaraneni S. Sirohi S. Dighe S. V. Walker E. A. Yoburn C. B. (2008). Hydromorphone efficacy and treatment protocol impact on tolerance and mu-opioid receptor regulation. *Eur. J. Pharmacol.* 3 39–45. 10.1016/j.ejphar.2008.08.025 18789923 PMC2586831

[B41] Leff E. R. Arttamangku S. Williams J. T. (2020). Chronic treatment with morphine disrupts kinase-dependent desensitization of GPCRs. *Mol. Pharmacol.* 4 497–507. 10.1124/mol.119.119362 32362586 PMC7562982

[B42] Levitt E. S. Williams J. T. (2012). Morphine desensitization and cellular tolerance are distinguished in rat locus coeruleus neurons. *Mol. Pharmacol.* 82 983–992. 10.1124/mol.112.081547 22914548 PMC3477235

[B43] Levitt E. S. Williams J. T. (2018). Desensitization and tolerance of mu opioid receptors on pontine kolliker-fuse neurons. *Mol. Pharmacol.* 93 8–13. 10.1124/mol.117.109603 29097440 PMC5708089

[B44] Llorente J. Lowe J. D. Sanderson H. S. Tsisanova E. Kelly E. Henderson G. (2012). μ-opioid receptor desensitization: homologous or heterologous? *Eur. J. Neurosci.* 36 3636–3642. 23002724 10.1111/ejn.12003 PMC3527680

[B45] Lowe J. D. Sanderson H. S. Cooke A. E. Ostovar M. Tsisanova E. Withey S. L. (2015). Role of G protein-coupled receptor kinases 2 and 3 in m-opioid receptor desensitization and internalization. *Mol. Pharmacol.* 88 347–356. 10.1124/mol.115.098293 26013542 PMC4518089

[B46] Madia P. A. Digne S. V. Sirohi S. Walker E. A. Yoburn B. C. (2009). Dosing protocol and analgesic efficacy determine opioid tolerance in the mouse. *Psychopharmacology* 3 413–422. 10.1007/s00213-009-1673-6 19816677

[B47] Matsui A. Jarvie B. C. Robinson B. G. Hentges S. T. Williams J. T. (2014). Separate GABA afferents to dopamine neurons mediate acute action of opioids, development of tolerance, and expression of withdrawal. *Neuron* 6 1346–1356. 10.1016/j.neuron.2014.04.030 24857021 PMC4072256

[B48] Melief E. J. Miyatake M. Bruchas M. R. Chavkin C. (2010). Ligand-directed c-Jun N-terminal kinas activation disrups opioid receptor signaling. *Proc. Natl, Acad. Sci. U.S.A* 107 11608–11613. 10.1073/pnas.1000751107 20534436 PMC2895055

[B49] Miess E. Gondin A. B. Yousuf A. Steinborn R. Mösslein N. Yang Y. (2018). Multisite phosphorylation is required for sustained interaction with GRKs and arrestins during rapid m-opioid receptor desensitization. *Sci. Signal.* 11:eaas9609. 10.1126/scisignal.aas9609 30018083

[B50] Paul A. K. Smith C. M. Rahmatullah M. Nissapaltom V. Wilairalana P. Spetea M. (2021). Opioid analgesic and opioid-induced adverse effects: a review. *Pharmaceuticals* 14:1091. 10.3390/ph14111091 PMC8620360 34832873

[B51] Pawar M. Kumar P. Sunkaraneni S. Sirohi S. Walker E. A. Yoburn B. C. (2007). Opioid agonist efficacy predicts the magnitude of tolerance and regulation of mu-opioid receptors and dynamin-2. *Eur. J. Pharmacol.* 3 91–101. 10.1016/j.ejphar.2007.01.059 17349996 PMC1995431

[B52] Pennock R. L. Dicken M. S. Hentges S. T. (2012). Multiple inhibitory G-protein-coupled receptors resist acute desensitization in the presynaptic but not postsynaptic compartments of neurons. *J. Neurosci.* 32 10192–10200. 10.1523/JNEUROSCI.1227-12.2012 22836254 PMC3418873

[B53] Quillinan N. Lau E. K. Virk M. von Zastrow M. Williams J. T. (2011). Recovery from mu-opioid receptor desensitization after chronic treatment with morphine and methadone. *J. Neurosci.* 31 4434–4443. 10.1523/JNEUROSCI.4874-10.2011 21430144 PMC3092436

[B54] Sharma S. K. Klee W. A. Nirenberg M. (1975). Dual regulation of adenylate cyclase accounts for narcotic dependence and tolerance. *Proc. Natl. Acad. Sci. U.S.A* 8 3092–3096. 10.1073/pnas.72.8.3092 1059094 PMC432926

[B55] Shoji Y. Delfs J. Williams J. T. (1999). Presynaptic inhibition of GABA-B-mediated synaptic potentials in the VTA during morphine withdrawal. *J. Neurosci.* 19 2347–2355. 10.1523/JNEUROSCI.19-06-02347.1999 10066284 PMC6782572

[B56] Sirohi S. Dighe S. V. Madia P. Yoburn B. C. (2009). The relative potency of inverse opioid agonists and a neutral opioid antagonist in precipitated withdrawal and antagonism of analgesia and toxicity. *J. Pharmacol. Expt. Ther.* 2 513–519. 10.1124/jpet.109.152678 19435929 PMC2713087

[B57] Stahl E. L. Bohn L. M. (2021). Low intrinsic efficacy alone cannot explain the improved side effect profiles of new opioid agonists. *Biochemistry* [Online ahead of print], 10.1021/acs.biochem.1c00466 34468132 PMC8885792

[B58] Terwilliger R. Z. Beitner-Johnson D. Sevarino K. A. Crain S. M. Nestler E. J. (1991). A general role for adaptations in G-proteins and the cyclic AMP system in mediating the chronic actions of morphine and cocaine on neuronal function. *Brain Res.* 2 100–110. 10.1016/0006-8993(91)91111-d 1651140

[B59] Uddin U. Jenne C. Fox M. E. Arakawa K. Keller A. Cramer N. (2021). Divergent profiles of fentanyl withdrawal and associated pan in mice and rat. *Pharmacol. Biochem. Behav.* 200:173077. 10.1016/j.pbb.2020.173077 33316293 PMC8065268

[B60] Vaughan C. W. Ingram S. L. Connor M. A. Christie M. J. (1997). How opioids inhibit GABA-mediated neurotransmission. *Nature* 390 611–614. 10.1038/37610 9403690

[B61] Virk M. S. Williams J. T. (2008). Agonist-specific regulation of mu-o[opioid receptor desensitization and recovery from desensitization. *Mol. Pharmcacol.* 4 1301–1308. 10.1124/mol.107.042952 18198283 PMC3640555

[B62] Walker A. E. Young A. M. (2001). Differential tolerance to antinociceptive effects of mu opioid during repeated treatment with etonitazene, morphine or buprenorphine in rats. *Psychopharmacology* 2 131–142. 10.1007/s002130000620 11314675

[B63] Whistler J. L. Chuang H. Chu P. Jan L. Y. von Zastrow M. (1999). Functional dissociation of μ opioid receptor signaling and endocytosis. *Neuron* 4 737–746. 10.1016/s0896-6273(01)80032-5 10482240

[B64] Whistler J. L. von Zastrow M. (1998). Morphine-activated opioid receptors elude desensitization by beta-arrestin. *Proc. Natl. Acad. Sci. U.S.A* 17 9914–9919. 10.1073/pnas.95.17.9914 9707575 PMC21436

[B65] Williams J. T. Christie M. Manzoni O. (2001). Cellular and synaptic adaptations mediating opioid dependence. *Physiol. Rev.* 81 299–343. 10.1152/physrev.2001.81.1.299 11152760

[B66] Williams J. T. Ingram S. J. Henderson G. Chavkin C. von Zastrow M. Schulz S. (2013). Regulation of μ-opioid receptors: desensitization, phosphorylation, internalization, and tolerance. *Pharmacol. Rev.* 65 223–254. 10.1124/pr.112.005942 23321159 PMC3565916

[B67] Zhang J. Ferguson S. S. G. Barak L. S. Bodduluri S. R. Laporte S. A. Law P. Y. (1998). Role of G protein-coupled receptor kinase in agonist-specific regulation of mu-opioid receptor responsiveness. *Proc. Natl. Acad. Sci. U.S.A* 12 7157–7162. 10.1073/pnas.95.12.7157 PMC22772 9618555

